# The Patterning and Proportion of Charged Residues in the Arginine-Rich Mixed-Charge Domain Determine the Membrane-Less Organelle Targeted by the Protein

**DOI:** 10.3390/ijms23147658

**Published:** 2022-07-11

**Authors:** Tamami Miyagi, Rio Yamazaki, Koji Ueda, Satoshi Narumi, Yuhei Hayamizu, Hiroshi Uji-i, Masahiko Kuroda, Kohsuke Kanekura

**Affiliations:** 1 Department of Molecular Pathology, Tokyo Medical University, 6-1-1 Shinjuku, Shinjuku-ku, Tokyo 160-8402, Japan; s121060@tokyo-med.ac.jp (T.M.); s122066@tokyo-med.ac.jp (R.Y.); 2 Cancer Proteomics Group, Cancer Precision Medicine Center, Japanese Foundation for Cancer Research, 3-8-31 Ariake, Koto-ku, Tokyo 135-8550, Japan; koji.ueda@jfcr.or.jp; 3 Department of Molecular Endocrinology, National Research Institute for Child Health and Development, 2-10-1 Okura, Setagaya-ku, Tokyo 157-8535, Japan; narumi-s@ncchd.go.jp; 4 Department of Materials Science and Engineering, School of Materials and Chemical Technology, Tokyo Institute of Technology, 2-12-1, Ookayama, Meguro-ku, Tokyo 152-8550, Japan; hayamizu.y.aa@m.titech.ac.jp; 5 Research Institute for Electronic Science (RIES), Hokkaido University, Kita 10 Nishi 20, Kita Ward, Sapporo 001-0020, Japan; hiroshi.ujii@es.hokudai.ac.jp; 6 Division of Information Science and Technology, Graduate School of Information Science and Technology, Hokkaido University, Kita 14 Nishi 9, Kita Ward, Sapporo 001-0020, Japan

**Keywords:** liquid–liquid phase separation, membrane-less organelle, nuclear speckle, nucleolus

## Abstract

Membrane-less organelles (MLOs) are formed by biomolecular liquid–liquid phase separation (LLPS). Proteins with charged low-complexity domains (LCDs) are prone to phase separation and localize to MLOs, but the mechanism underlying the distributions of such proteins to specific MLOs remains poorly understood. Recently, proteins with Arg-enriched mixed-charge domains (R-MCDs), primarily composed of R and Asp (D), were found to accumulate in nuclear speckles via LLPS. However, the process by which R-MCDs selectively incorporate into nuclear speckles is unknown. Here, we demonstrate that the patterning of charged amino acids and net charge determines the targeting of specific MLOs, including nuclear speckles and the nucleolus, by proteins. The redistribution of R and D residues from an alternately sequenced pattern to uneven blocky sequences caused a shift in R-MCD distribution from nuclear speckles to the nucleolus. In addition, the incorporation of basic residues in the R-MCDs promoted their localization to the MLOs and their apparent accumulation in the nucleolus. The R-MCD peptide with alternating amino acids did not undergo LLPS, whereas the blocky R-MCD peptide underwent LLPS with affinity to RNA, acidic poly-Glu, and the acidic nucleolar protein nucleophosmin, suggesting that the clustering of R residues helps avoid their neutralization by D residues and eventually induces R-MCD migration to the nucleolus. Therefore, the distribution of proteins to nuclear speckles requires the proximal positioning of D and R for the mutual neutralization of their charges.

## 1. Introduction

Eukaryotic cells carry out a wide variety of biochemical reactions simultaneously. Some of these reactions lead to toxic byproducts and are mutually incompatible. Hence, these biochemical reactions occur in parallel in isolated compartments in typical lipid-membrane-bound organelles. In contrast, cells form dynamic ribonucleoprotein compartments to respond rapidly to various external and internal stimuli [1,2]. These ribonucleoprotein bodies, formed through the liquid–liquid phase separation (LLPS) of proteins and RNA, are known as membrane-less organelles (MLOs) [3]. MLOs such as nuclear speckles, stress granules, and the nucleolus are formed according to the requirements of the cell and are eliminated when unused. These structures can change their size, fluidity, and protein composition according to requirements, to mediate a dynamic cellular response. The most important factor in distinguishing between membrane-bound organelles and MLOs is the ability of material to move freely across the boundaries of the organelle. This is attributed to the presence or absence of a lipid membrane. In addition, due to this feature, disease-associated proteins such as amyotrophic lateral sclerosis (ALS)-causing *C9ORF72* dipeptides are often incorporated into MLOs and influence their dynamics [4]. MLOs have constituent proteins with low-complexity domains (LCDs) and/or intrinsically disordered regions (IDRs). The repetitive motifs of LCDs promote multivalent interaction, and the flexibility of IDRs makes them strong driving factors of phase separation [5,6]. Multivalent interactions occurring through LCD-IDRs promote the polymerization of macromolecules, leading to phase separation and MLO formation. However, the process of distribution of MLO-targeting proteins to specific MLOs remains elusive.

The localization of molecules to the nucleolus is the most widely studied process in MLO targeting. The consensus regarding the nucleolus localization signals (NoLS) is a sequence with a cluster of basic amino acids such as arginine (R) and lysine (K) [7,8]. Findings from the comparison of various NoLS and proteomic studies on the nucleolus suggest that NoLS do not contain any specific sequences that act as a recognition motif for binding to specific receptor molecules [9,10]; instead, they undergo high-affinity interactions with nucleolar molecules such as proteins and nucleic acids, thus facilitating nucleolar localization. However, the precise mechanism remains unknown. The targeting of nuclear speckles by proteins has also been investigated. A nuclear speckle is an MLO with an essential role in mRNA splicing, the splicing factor storage, and modification [11]. SON protein and serine/arginine repetitive matrix protein 2 (SRRM2), both natively unfolded proteins with large LCDs, have been identified as the scaffolding molecules for nuclear speckles [12]. Recently, the R-enriched mixed-charge domain (R-MCD) was shown to determine whether proteins will localize to nuclear speckles [13]. Interestingly, the combination of aspartate (D) and R in proteins triggers their incorporation into nuclear speckles, whereas the presence of glutamate (E) and K exerts a limited effect on protein localization. However, the role of the R-MCD structure–function relationship in protein distribution to nuclear speckles is yet to be fully characterized.

In this study, we first tested a series of artificial R-MCD variants with different periodicities or different proportions to determine how the charge position and net charge affect the distribution of proteins to the MLOs. When the positive and negative charges were separated, the localization of R-MCDs shifted from nuclear speckles to the nucleolus, even though the net charge remained neutral. Furthermore, we showed that the incorporation of R-MCD into MLOs was enhanced when their net charge was positive. We performed interactome analysis by proximity-dependent biotin labeling and showed that proteins from the nucleolar granular components (GCs) were enriched in the interactome of R-MCD with blocky positive/negative charges. Lastly, the R-MCD peptide with opposite charges segregated was found to be susceptible to phase separation and associated with RNA, poly-E peptide, and nucleophosmin (NPM1), a nucleolar protein. These results indicate that the net charge of the LCD and the position of the charged amino acids in the LCD determine the degree of phase separation and the localization of proteins to specific MLOs.

## 2. Results

### 2.1. Charge Patterning in the R-MCDs Influences Their Distribution to MLOs

**Figure 1:**
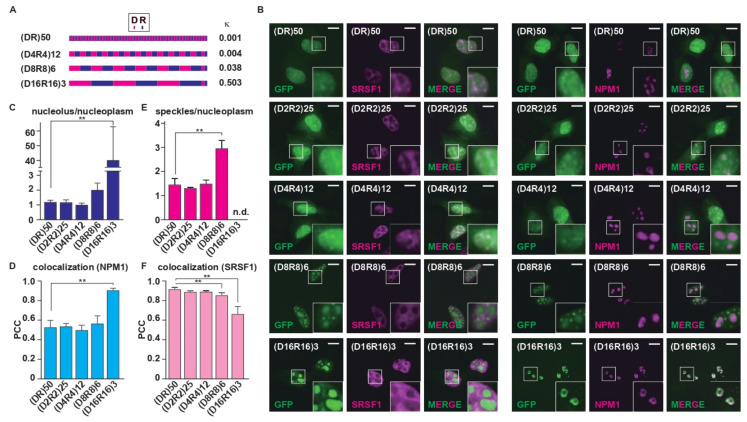
The periodicity of the (DR)_50_ repeat determines the MLOs targeted by the R-MCD. (**A**) Schematic structures of (DR)_50_ variants with different repeat periodicities. D_2_R_2_ was fused to the C-terminus of (D_4_R_4_)_12_, (D_8_R_8_)_6_, and (D_16_R_16_)_3_ to equalize the number of D and R residues. Kappa (κ: charge patterning parameter) was calculated by CIDER [6]. (**B**) Subcellular localization of GFP-fused R-MCD variants in HeLa cells. Nuclear speckles were visualized by co-expressed serine/arginine-rich splicing factor 1 (SRSF1)-mCherry, and the nucleolus was visualized by co-expressed nucleophosmin (NPM1)-DsRed. Scale bar = 10 µm. The inset images are high-magnification images of squared areas. (**C**) Ratio of GFP signals in the nucleolus/nucleoplasm, with *n* = 8~10 cells/condition. (**D**) The degree of colocalization between GFP-(DR)_50_ variants and NPM1 quantified by the Pearson correlation coefficient (PCC). (**E**) The ratio of GFP signals in nuclear speckles/nucleoplasm. The ratio for (D_16_R_16_)_3_ was not determined because only a few cells showed speckle incorporation. *N* = 8~10 cells/condition. (**F**) The degree of colocalization between GFP-(DR)_50_ variants and SRSF1, quantified by the PCC. The error bars show ± SD. The asterisks indicate significant differences obtained via one-way analysis of variance (ANOVA) with Dunnett’s test; ** *p* < 0.01.

**Figure 2:**
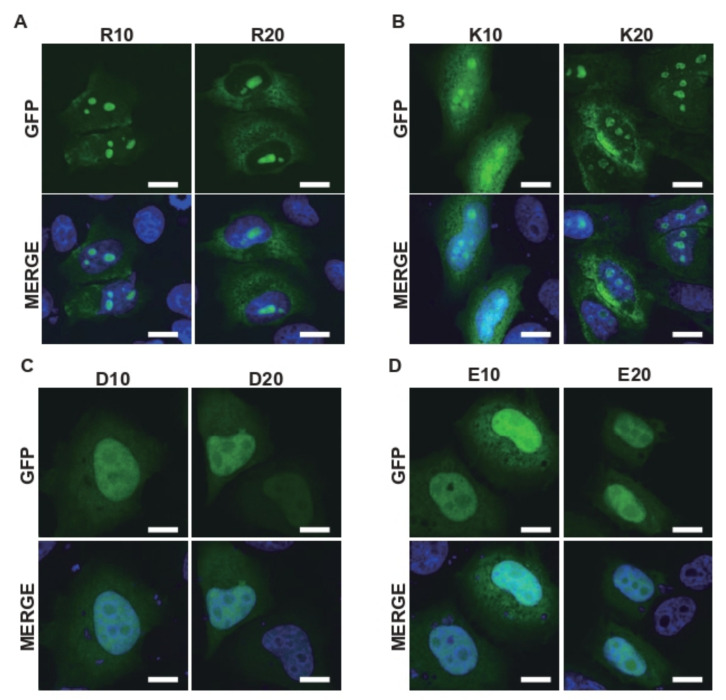
The presence of consecutive basic amino acids is responsible for the nucleolar distribution of proteins. (**A**–**D**) The subcellular localization of ten or twenty consecutive basic amino acids (R for (**A**) and K for (**B**)) or acidic amino acids (D for (**C**) and E for (**D**)) fused to the C-terminus of GFP expressed in HeLa cells. Scale bar = 10 µm.

To investigate how the position of oppositely charged residues in the R-MCDs affects their subcellular localization, we synthesized (DR)_50_ variants with different charge distributions as models for R-MCD (Figure 1A). All (DR)_50_ variants carried the same number of R (*n* = 50) and D (*n* = 50) residues, and the charge patterning was the only difference, as indicated by the charge patterning parameter κ (Figure 1A and Figure S1) [14]. As previously reported, when overexpressed in HeLa cells, green fluorescent protein (GFP)-(DR)_50_ colocalized with serine/arginine-rich splicing factor 1 (SRSF1), a nuclear speckle marker (Figure 1B) [15]. When we changed the charge patterning without changing the net charge, (D_8_R_8_)_6_ tended to accumulate in the nucleolus, and (D_16_R_16_)_3_ localized almost exclusively to the nucleolus, as shown by a good Pearson correlation coefficient (PCC) with a nucleolar marker, NPM1-DsRed [16] (Figure 1B–F). Reportedly, the localization of proteins to the nucleolus requires the presence of basic amino acids such as R and K at high levels [7,8,9]. However, in the case of (DR)_50_ variants, even though both (DR)_50_ and (D_16_R_16_)_3_ had the same number of D and R residues and equal net charges, (D_16_R_16_)_3_ migrated to the nucleolus, whereas (DR)_50_ accumulated in nuclear speckles. This suggests that uneven intramolecular charge distribution may be a key determinant of protein localization to MLOs. To elucidate the roles of the acidic and basic blocks of (D_16_R_16_)_3_ in the nucleolar localization, we tested the localization of contiguous acidic and basic amino acids. When we overexpressed R_10_, R_20_, K_10_, or K_20_, contiguous basic amino acid chains of different lengths showed nucleolar localization (Figure 2A,B). However, D_10_, D_20_, E_10_, or E_20_ expressed in HeLa cells showed no specific localization to MLOs (Figure 2C,D), suggesting that the localization of (D_16_R_16_)_3_ to the nucleolus is owing to the presence of contiguous basic amino acids. These results indicate that the primary sequence of oppositely charged residues determines the MLO protein targets.

### 2.2. Charge Patterning Affects the Interactome of R-MCDs

**Figure 3:**
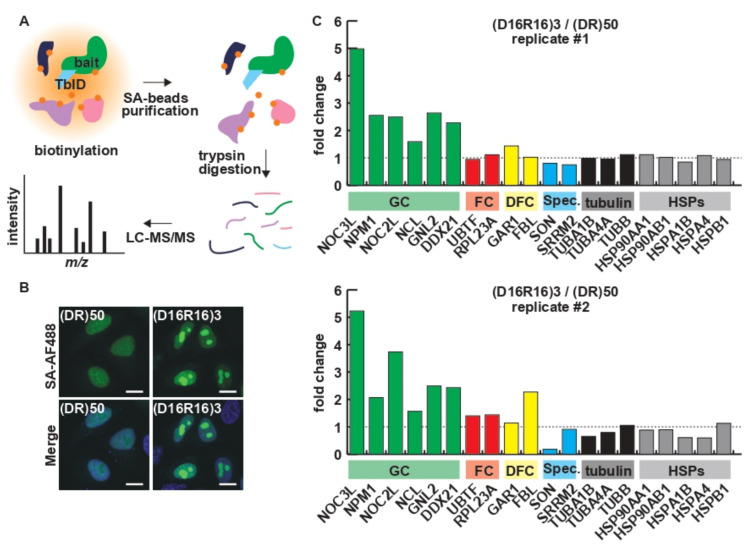
Proximity labeling analysis revealed that charge patterning affects the interactome of R-MCDs. (**A**) Schematic illustration of TurboID (TbID)-mediated proximity biotin labeling. (DR)_50_ or (D_16_R_16_)_3_ was used as the bait in the experiment. SA: streptavidin. (**B**) Protein biotinylation by TurboID-(DR)_50_ or TurboID-(D_16_R_16_)_3_, followed by visualization with AlexaFluor488-labeled streptavidin (SA-AF488). Scale bar = 10 µm. (**C**) Fold change in the signals of markers for the granular component (GC), fibrillar center (FC), or dense fibrillar component (DFC) of the nucleolus, nuclear speckles (Spec.), tubulin species, and heat-shock proteins (HSPs). Proximity labeling analysis followed by liquid chromatography–tandem mass spectrometry (LC–MS/MS) was performed twice for independently prepared sample sets.

As indicated by the incorporation of (DR)_50_ into the nuclear speckle and the localization of (D_16_R_16_)_3_ to the nucleolus, charge patterning affects the protein–protein interaction of R-MCDs. Since R and D are highly charged residues, conventional immunoprecipitation/proteomics analysis, which requires cell lysis, may lead to artificial interactions during the lysis/immunoprecipitation process. To investigate the protein–protein interactions while maintaining spatial information, we performed TurboID-mediated proximity biotin labeling of the proteomes in close proximity to each R-MCD (Figure 3A) [17,18]. The visualization of biotinylated proteins using AlexaFluor488-conjugated streptavidin revealed that TurboID-(DR)_50_ exclusively biotinylated proteins in nuclear speckles, and TurboID-(D_16_R_16_)_3_ biotinylated the proteins in the nucleolus, as expected (Figure 3B). Biotinylated proteomes were analyzed using quantitative liquid chromatography–mass spectrometry (LC–MS) [19]. Signals from nucleolar markers and speckle markers showed that the segregation of oppositely charged residues in the R-MCD increased the cohesion of the nucleolar GC, where rRNA and proteins assemble in the ribosomal subunit, and reduced the cohesion to speckle markers (Figure 3C). Some nuclear and cytoplasmic MLOs have been shown to require RNA to maintain their integrity [20]. RNA has a negative charge and can interact with basic amino acids via electrostatic interactions, and RNA depletion by intranuclear expression of RNAse induces morphological changes in MLOs [20]. Furthermore, the inhibition of RNA transcription by actinomycin D (ActD) is known to cause the enlargement of nuclear speckles and dwarfing of the nucleolus [21,22,23]. A decrease in the levels of negatively charged RNAs alters the subcellular localization of proteins with a high basic amino acid content [24]. We investigated how the ActD-mediated inhibition of RNA synthesis affects the localization of R-MCD. The distribution of (DR)_50_, which localizes to nuclear speckles, showed no significant change in colocalization with SRSF1 after ActD treatment (Figure S2A–C). However, the distribution of (D_16_R_16_)_3_, which localizes to the nucleolus, was significantly affected upon ActD treatment and showed a granular pattern in the nucleoplasm (Figure S2D–F), suggesting that electrostatic interaction of (D_16_R_16_)_3_ with RNA may be one of the drivers of its distribution to the nucleolus.

### 2.3. A Net Positive Charge in R-MCDs Enhances Their Incorporation into MLOs

**Figure 4:**
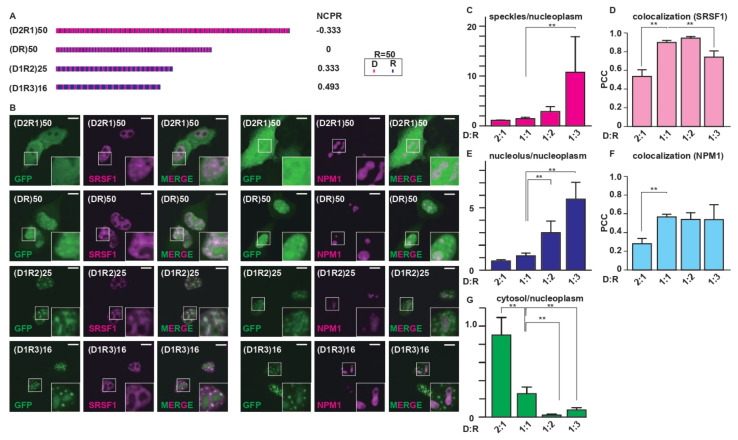
Imparting a net positive charge to the R-MCD enhances its ability to target MLOs. (**A**) Schematic structures of (DR)_50_ proportional variants with different net charges per residue (NCPR) [6]. (**B**) Subcellular localization of GFP-fused (DR)_50_ proportional variants in HeLa cells. Nuclear speckles were visualized by co-expressed SRSF1-mCherry, and the nucleolus was visualized by co-expressed NPM1-DsRed. Scale bar = 10 µm. The inset images are high-magnification images of squared areas. (**C**) The ratio of GFP signals in nuclear speckles/nucleoplasm, with *n =* 10~12 cells/condition. (**D**) The degree of colocalization between GFP-(DR)_50_ proportional variants and SRSF1 quantified by PCC. (**E**) The ratio of GFP signals in the nucleolus/nucleoplasm, with *n* = 10~12 cells/condition. (**F**) The degree of colocalization between GFP-(DR)_50_ proportional variants and NPM1 quantified by PCC. (**G**) The ratio of GFP signals in the cytosol/nucleoplasm, with *n* = 10~12 cells/condition. The error bars show ± SD. The asterisks indicate significant differences derived using one-way ANOVA with Dunnett’s test; ** *p* < 0.01.

Next, we examined how the net charge of an R-MCD affects its subcellular localization. To this end, we synthesized R-MCD variants containing the same number of R residues (*n* = 50) with different net charges (Figure 4A). The net charge per residue (NCPR) of these R-MCD variants ranged from −0.333 to 0.493. An increase in the NCPR through R up to +0.2 was shown to enhance the incorporation of (DR)_50_ mutants into nuclear speckles [13]. GFP-(D_2_R_1_)_50_ (NPCR = −0.333) showed a diffused distribution pattern and low cohesion with nuclear speckles (Figure 4B). When the proportion of R-MCD changed and the number of acidic amino acids was reduced to impart a net positive charge, the condensation of R-MCDs increased not only in nuclear speckles but also in the nucleolus (Figure 4B–G). This effect was more evident when the net charge increased, which suggests that an increase in the net positive charge of R-MCDs enhances their condensation in MLOs (Figure 4C,E). Note that when the net charge of R-MCDs was negative, their nuclear localization ceased and they diffused into the nucleus and cytoplasm (Figure 4G).

### 2.4. Charge Patterning in Natural R-MCDs Determines Protein Distribution to Specific MLOs

**Figure 5:**
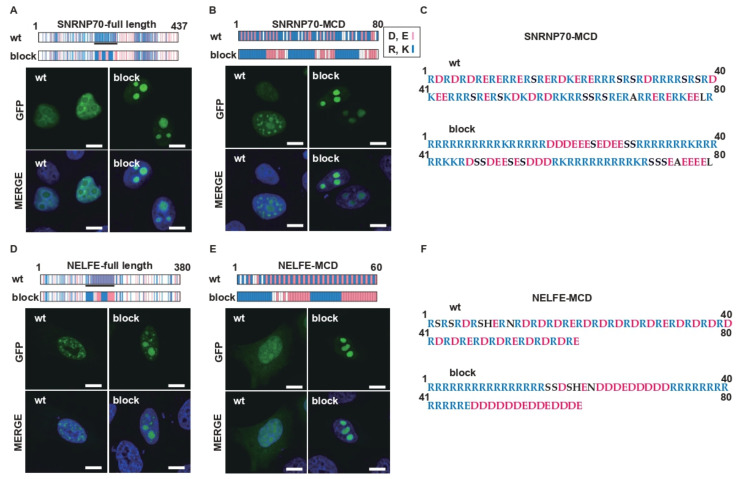
Charge patterning in a natural R-MCD determines the distribution of proteins to specific MLOs. (**A**) The schemes (top) show the structures of wild-type (wt) full-length SNRNP70 or full-length SNRNP70 with the R-MCD of blocky charges (block). The pink lines indicate acidic amino acids (D and E), and the blue lines indicate basic amino acids (R and K). The black line represents the R-MCD analyzed in this study. The lower panels show the localization of GFP-wt-SNRNP70 or GFP-block-SNRNP70. (**B**) The structure and subcellular localization of the wt-R-MCD of SNRNP70 or the block-R-MCD of SNRNP70. (**C**) The amino acid sequence of the wt or block of SNRNP70-MCD. (**D**) The structure and subcellular localization of wt full-length NELFE (wt) or full-length NELFE with the block-R-MCD (block). (**E**) The structure and subcellular localization of the wt-R-MCD of NELFE or the block-R-MCD of NELFE. (**F**) The amino acid sequence of the wt or block of NELFE-MCD. Scale bar = 10 µm.

Next, we tested the effect of charge patterning on the R-MCDs of natural nuclear speckle-associated proteins. Wild-type (wt) full lengths of small nuclear ribonucleoprotein U1 subunit 70 (SNRNP70-FL) and wt-SNRNP70-MCD were localized to nuclear speckles (Figure 5A–C). When oppositely charged amino acids were segregated without changing the net charge, the site of localization shifted from the nuclear speckles to the nucleolus. Similarly, the site of localization of the full-length protein and MCD of negative elongation factor complex member E (NELFE) also changed from the nuclear speckles to the nucleolus when the distribution of charged amino acids was changed (Figure 5D–F). These results show that the charge patterning effects observed in the artificial R-MCD are similar to those in natural proteins and are a means of controlling the localization of proteins to MLOs in cells.

### 2.5. Charge Patterning Determines the Phase-Separating Properties of R-MCD Peptides

**Figure 6:**
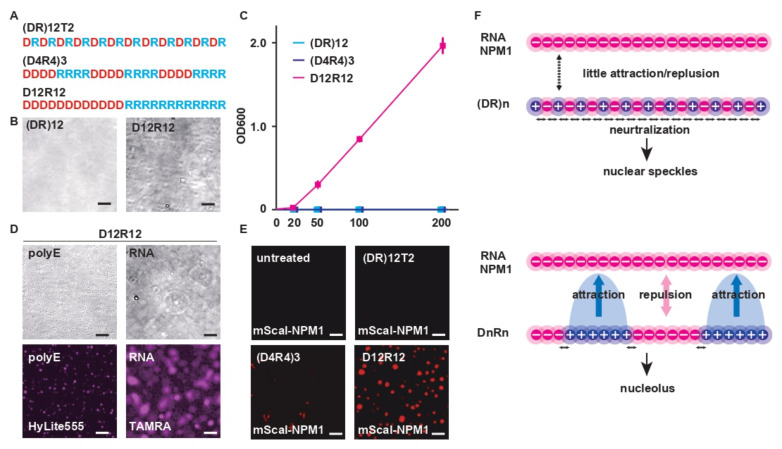
Charge patterning determines the phase-separation properties of R-MCD peptides. (**A**) The structure of (DR)_12_ variants with different periodicities. (**B**) D_12_R_12_, but not (DR)_12_, underwent simple coacervation. Scale bar: 10 µm. (**C**) The turbidity of (DR)_12_ variants with different periodicities at different concentrations determined using the OD_600_, with *n* = 3. The error bars show ± SD. (**D**) The D_12_R_12_ droplets mixed with 10 nM TAMRA-labeled rA15 RNA or 10 ng/µL of HyLite555-labeled polyE peptide. (**E**) Recombinant mScarlet-I-NPM1 protein mixed with each (DR)_12_ variant peptide (10 µM). (**F**) Scheme of intramolecular neutralization by oppositely charged amino acids in the vicinity of (DR)n and DnRn.

The molecular mechanism by which charge segregation in the MCD causes a shift in localization from nuclear speckles to the nucleolus remains unknown. We synthesized (DR)_12_ mutants with different charge patterns and tested their biochemical characteristics with respect to phase separation (Figure 6A). Among the mutants, D_12_R_12_ underwent simple coacervation and formed droplets (Figure 6B). (DR)_12_ and (D_4_R_4_)_3_ did not undergo phase separation, even when their concentrations were increased (Figure 6C). When a fluorescently labeled polyE peptide or RNA (rA15) was added to the droplets formed by D_12_R_12_, these acidic molecules were incorporated into the droplets (Figure 6D). Furthermore, when we added the (DR)_12_ mutants to recombinant NPM1 protein, a nucleolar protein with acidic motifs, D_12_R_12_ underwent phase separation, but other peptides did not (Figure 6E). Thus, the positioning of opposite charges in an alternating pattern in the R-MCDs causes mutual charge neutralization and leads to the loss of affinity of R-MCDs for neighboring molecules. The marginal unevenness in local charge is important for the localization of proteins to nuclear speckles (Figure 6F). However, the segregation of oppositely charged residues reduces the likelihood of neutralization of positive and negative charges, and as a result, positively charged amino acids may bind to RNAs in a loop-like structure and localize to the nucleolus. To prove that the electrostatic force is retained more strongly in (D_16_R_16_)_3_ compared with (DR)_50_, we challenged the HeLa cells expressing GFP-(DR)_50_ or GFP-(D_16_R_16_)_3_ with a digitonin solution containing different concentrations of NaCl. After cell permeabilization, (D_16_R_16_)_3_ localized to the nucleolus even in the presence of 200 mM NaCl, but (DR)_50_ lost its localization to nuclear speckles in the presence of 100 mM NaCl, suggesting that (D_16_R_16_)_3_ binds to the surrounding molecules via stronger electrostatic forces than (DR)_50_ (Figure S3A,B).

## 3. Discussion

MLOs form and disappear dynamically in the nucleus and cytoplasm and play essential roles in cellular homeostasis. The appropriate distribution of each MLO-specific component to the designated MLOs is essential for the maintenance of their functions, but the mechanism underlying their distribution is unknown. In this study, we showed that the charge pattern in R-MCD determines the phase separation of proteins to MLOs and that segregation of residues with opposite charges influences the interactome of R-MCD, promoting phase separation and shifting their distribution from nuclear speckles to the nucleolus.

The (DR)_50_ variants with different periodicities used in this study were fused with enhanced GFP and had an isoelectric point of 5.6. Although EGFP and EGFP-(DR)_50_, with different periodicities, have similar isoelectric points (pI = 5.6), the EGFP-(DR)_50_ variants are localized to nuclear speckles or the nucleolus. Thus, even though the net charge of the R-MCD is zero, the abundance of basic amino acids acts as a nuclear-localizing signal and induces nuclear protein distribution.

Nuclear speckles are the nuclear MLOs with an important role in mRNA splicing, storage, maturation, and splicing-factor modification [25]. The mechanism of nuclear speckle formation remains unknown. Recently, SRRM2 and SON were identified as speckle scaffold proteins [12]. While SRRM2 and SON were shown to be essential for nuclear speckle formation, the process by which SRRM2 and SON form nuclear speckles has not been determined. Many splicing factors that localize to nuclear speckles harbor D/R or S/R repeats [26,27,28]. The D/R and S/R repeats are reportedly essential for the localization of factors to nuclear speckles [13]. Poly(PR) and poly(GR) dipeptides produced from the mutant *C9ORF72* gene, which is associated with ALS [29,30], are structurally similar to (DR)_50_ in that they contain alternating R residues; however, their net charge is positive and their targeted MLO is the nucleolus [31], suggesting that the neutralization of the charge on R by neighboring acidic amino acids is important for the localization of factors to nuclear speckles. This is consistent with the fact that when phosphorylated, serine residues in the S/R repeat sequence act as acidic amino acids, to neutralize R-MCDs and induce their localization to nuclear speckles [13].

Peptides with an alternating sequence of acidic and basic amino acids are known as zwitterionic peptides. Zwitterionic peptides are easily hydrated and interact poorly with proteins and charged molecules [32,33]. Because many strongly charged molecules such as RNA are present in the nucleus, natural R-MCDs use their D/R-alternating zwitterionic structure to reduce their susceptibility to the strong attractive force of RNA and localize to nuclear speckles. Conversely, once the oppositely charged residues are segregated and the local charge unevenness is established, the zwitterionic peptide loses its properties and accumulates in the nucleolus by changing its molecular binding mode. Although both the nucleolus and nuclear speckle contain RNA, the nucleolus contains much more abundant RNA than the nuclear speckles, as shown by strong signals when intracellular RNA is stained [34], and the abundant RNA-derived electrostatic forces might attract the blocky charged sequences, which (DR)_50_ can escape. On the other hand, the mechanism of how (DR)_50_ specifically condenses to nuclear speckles remains unclear. The zwitterionic structure may be advantageous for specific interaction with SON, SRRM2, or other components of nuclear speckles. The intra-organellar environmental differences such as hydrophobicity and density of aromatic compounds might also have an influence. Supporting the idea that the (D_16_R_16_)_3_ has stronger electrostatic interactions than (DR)_50_, (D_16_R_16_)_3_ remained in the nucleolus when cells were permeabilized with digitonin and treated with salts that shield electrostatic forces, whereas (DR)_50_ lost its localization to the nuclear speckle (Figure S3). Nevertheless, the possibility that blocked charged residues localize to the nucleolus by interactions with specific nucleolar molecules cannot be ruled out. It also remains unclear why a net positive charge in (DR)_50_ variants enhances their incorporation into nuclear speckles as well as the nucleolus. The NCPR of (D_1_R_3_)_50_ is 0.493 (Figure S1), which is similar to that of the ALS-causing *C9ORF72*-encoded R-rich dipeptides, poly(GR) and poly(PR) (NPCR = 0.5 for both), which exclusively localize to the nucleolus [31]. Therefore, we believe that the positioning of the anionic D residue next to the cationic R residue is necessary for protein localization to the speckle; however, further studies are warranted.

Only few studies have reported the role of the charge pattern of R-MCD in the protein localization to MLOs. Greig et al. reported that modest unevenness of charge distribution in the R-MCD enhances cohesion for both the nucleolus and nuclear speckles, and also a shift in the net charge of R-MCD to positive increases the size of the nuclear speckle and triggers retention of mRNA in the nucleus [13]. Therefore, the charge distribution of the R-MCD plays an important role not only in the subcellular localization but also in the protein function.

Research on the mechanism of localization to each MLO has only commenced recently. MLOs are involved in various intracellular functions, and their disruption has been implicated in various diseases such as neurodegenerative diseases and malignant tumors. However, only a few drugs target MLOs. Klein et al. recently showed that small molecules such as cisplatin are incorporated into the droplets of super-enhancers; however, their controlled delivery to specific MLOs is yet to be achieved [35]. The targeting of specific MLOs by charge patterning, as shown here, may help develop novel drug delivery systems (DDS) in the future.

In conclusion, we found that charge patterning is important for the targeted distribution of proteins in specific MLOs and that it regulates the protein interactome as well as the manner of phase separation in MCDs. We believe that these data are of significance from both biological and medical perspectives and will help develop DDS that can target specific MLOs.

## 4. Materials and Methods

### 4.1. Recombinant Plasmids Construction

All MCD constructs and cDNAs for SNRNP70 (full length and MCD), NELFE (full length and MCD), and TurboID were synthesized and subcloned into pcDNA3.1-N-eGFP vector or pcDNA3.1 vector (for TurboID) by GenScript (Piscataway, NJ, USA). NPM1-DsRed was a gift from Mary Dasso (Addgene plasmid # 34,553; http://n2t.net/addgene:34553 (accessed on 6 July 2022); RRID:Addgene_34553). For bacterial expression, human NPM1 tagged with mScarlet-I cDNA was synthesized and subcloned into pET28a vector by GenScript. Human SRSF1 cDNA was PCR-amplified from a HeLa-cell cDNA pool with a sense primer (GGATCCATGTCGGGAGGTGGTGTG) and an antisense primer (GAATTCTTATGTACGAGAGCGAGATCTG) using PrimeSTAR Max DNA polymerase (Takara Bio, Tokyo, Japan) and subcloned into pmCherry-C1 vector (Clontech, Mountain View, CA, USA).

### 4.2. Transfection of HeLa Cells and Image Acquisition by Confocal Microscopy

HeLa cells (RIKEN BRC, Tsukuba, Japan) plated on a chambered glass slide (Matsunami, Osaka, Japan) were transfected with each GFP-MCD construct in the presence or absence of an MLO marker using Lipofectamine 2000 (Thermo Fisher Scientific, Waltham, MA, USA), in accordance with the manufacturer’s instructions. Twenty-four hours after transfection, the cells were fixed with formalin-PBS, and coverslips were mounted using ProLong Gold-DAPI antifade reagent (Thermo Fisher Scientific). For ActD treatment, the transfected HeLa cells were treated with 100 nM of ActD (Sigma-Aldrich, St. Louis, MO, USA) for the indicated period. For the digitonin permeabilization assay, the transfected HeLa cells were treated with a buffer containing 10 mM HEPES (pH 7.4), 0.1% digitonin (Nacalai, Kyoto, Japan), 300 mM sucrose, and 100 mM or 200 mM NaCl for 10 min before fixation. The cells were imaged with FV10i confocal microscopy (Olympus, Tokyo, Japan). Image analyses were performed using the ImageJ Fiji software (http://rsbweb.nih.gov/ij, accessed on 6 July 2022).

### 4.3. Peptide Synthesis and Purification of Recombinant mScarletI-NPM1

All (DR)_12_ variant peptides were synthesized by GenScript. Trifluoroacetic acid was substituted with acetic acid. For the expression of the recombinant mScarlet-I-NPM1 protein, *Escherichia coli* BL21 cells were transformed with pET28a-mScarlet-I-NPM1, and expression of NPM1 was induced by incubation of the cells in 1mM IPTG for 16 h at 25 °C. The recombinant protein was purified using a HisTalon gravity column (Clontech) and dialyzed with Tris-buffered saline.

### 4.4. Phase Separation of Peptides

The (DR)_12_ or D_12_R_12_ peptide (1 mM) was dissolved in water and diluted in a phase separation buffer (composed of 10 mM HEPES (PH7.4) and 100 mM NaCl) at 100 µM. The droplets were observed by FV10i confocal microscopy, and the OD_600_ value was measured using a NanoDrop One (Thermo Fisher). The droplets were mixed with 10 nM TAMRA-labeled rA15 RNA (Fasmac, Kanagawa, Japan) or 10 ng/µL of HyLite555-labeled polyE peptide [34]. For phase separation with mScarlet-I-NPM1, 1 µM mScarlet-I-NPM1 was mixed with each (DR)_12_ variant peptide (10 µM) in the phase separation buffer.

### 4.5. Proximity Labeling and Quantitative LC–MS

TurboID-mediated proximity labeling was performed as previously described [17]. Briefly, HeLa cells plated on a 15 cm dish were transfected with TurboID-(DR)_50_ or TurboID-(D_16_R_16_)_3_. Twenty-four hours after transfection, the cells were incubated with 50 µM biotin for 2 h. After the cells were washed four times with ice-cold PBS, they were lysed using RIPA buffer containing cOmplete protease inhibitor cocktail (Roche). Excess biotin was removed by ultrafiltration using an Amicon Ultra filter. For purifying biotinylated proteins, cell lysates were incubated with Dynabeads MyOne streptavidin beads (Thermo Fisher Scientific) in PBS for 2 h. The beads were sequentially washed with RIPA buffer, 1 M KCl, 0.1 M Na_2_CO_3_, 2 M urea in 10 mM Tris-HCl (pH 7.5), and RIPA buffer. The proteins were eluted by boiling with 2× Laemmli sample buffer (Bio-Rad, Hercules, CA, USA) and subjected to quantitative LC–MS [19]. Briefly, the extracted samples were reduced with 10 mM TCEP at 100 °C for 10 min, alkylated with 50 mM iodoacetamide at room temperature for 45 min, and separated using SDS-PAGE. Protein bands, visualized via Coomassie brilliant blue staining, were excised, destained, and cut finely before in-gel digestion with Trypsin/Lys-C Mix (Promega) at 37°C for 12 h. The digested peptides were analyzed with an Orbitrap Fusion Lumos mass spectrometer (Thermo Fisher Scientific) in combination with an UltiMate 3000 RSLC nano-flow HPLC system (Thermo Fisher Scientific) in the HCD MS/MS mode. Peptides were identified and quantified using Proteome Discoverer 2.4 software (Thermo Fisher Scientific), where the MS/MS spectra were searched against the *Homo sapiens* protein database in SwissProt (https://www.uniprot.org/, accessed on 6 July 2022), with a false discovery rate of 1% as an identification cutoff.

### 4.6. Colocalization Analysis

The colocalization analysis of GFP-(DR)_50_ variants with NPM1 or SRSF1 was performed with Fiji ImageJ software using the EzColocalization plugin [36], and the PCC for each combination was calculated.

### 4.7. Bioinformatic Analysis of Biochemical Properties of Peptides and Proteins

The biochemical properties of peptides and proteins used in this study were analyzed using the CIDER web server (http://pappulab.wustl.edu/CIDER/analysis/, accessed on 6 July 2022) [6]. The following components were analyzed and are shown in Figure S1: FCR, fraction of charged residues; NCPR, net charge per residue; Kappa (κ), charge patterning parameter; and hydropathy, the Kyte–Doolittle hydropathy score for the sequence on a scale of 0–9.

### 4.8. Statistic Analysis

Data are represented as means ± standard deviations. Statistical analysis of the data was performed using SPSS software 28 (IBM).

## Data Availability

The raw data supporting the conclusions of this article will be provided by the authors without undue reservation.

## Supplementary Materials

The following supporting information can be downloaded at: https://www.mdpi.com/article/10.3390/ijms23147658/s1.
