# Supplementary material for: The Patterning and Proportion of Charged Residues in the Arginine-Rich Mixed-Charge Domain Determine the Membrane-Less Organelle Targeted by the Protein
Source: Int J Mol Sci. 2022 Jul 11;23(14):7658. doi: 10.3390/ijms23147658 (PMC9324279; doi:10.3390/ijms23147658)
Supplement: Supplementary file 1 [file ijms-23-07658-s001.zip › Miyagi DR IJMS Fig.S1 053122.pdf]

Supplementary Figure S1

name	length	κ	FCR	NCPR	hydropathy
(DR)50	100	0.001	1.000	0.000	0.500
(D2R2)25	100	0.004	1.000	0.000	0.500
(D4R4)12	100	0.038	1.000	0.000	0.500
(D8R8)6	100	0.503	1.000	0.000	0.500
(D16R16)3	100	0.776	1.000	0.000	0.500
(D2R1)50	150	0.016	1.000	-0.333	0.667
(D1R2)25	75	0.016	1.000	0.333	0.333
(D1R3)16	67	0.047	1.000	0.493	0.254
U1-70K full wt	437	0.110	0.478	0.043	2.660
U1-70K full mut	437	0.312	0.478	0.043	2.660
U1-70K-MCD wt	80	0.084	0.850	0.275	0.977
U1-70K-MCD mut	80	0.700	0.850	0.275	0.977
NELFE full wt	380	0.161	0.387	0.024	3.227
NEFLE full mut	380	0.447	0.387	0.024	3.227
NEFLE-MCD wt	60	0.006	0.917	0.050	0.657
NEFLE-MCD mut	60	0.784	0.917	0.050	0.657
(DR)12	24	0.001	1.000	0.000	0.500
(D4R4)3	24	0.048	1.000	0.000	0.500
D12R12	24	1.000	1.000	0.000	0.500
