# Supplementary figures and images for: The Patterning and Proportion of Charged Residues in the Arginine-Rich Mixed-Charge Domain Determine the Membrane-Less Organelle Targeted by the Protein
Source: Int J Mol Sci. 2022 Jul 11;23(14):7658. doi: 10.3390/ijms23147658 (PMC9324279; doi:10.3390/ijms23147658)

Supplementary Figure S2

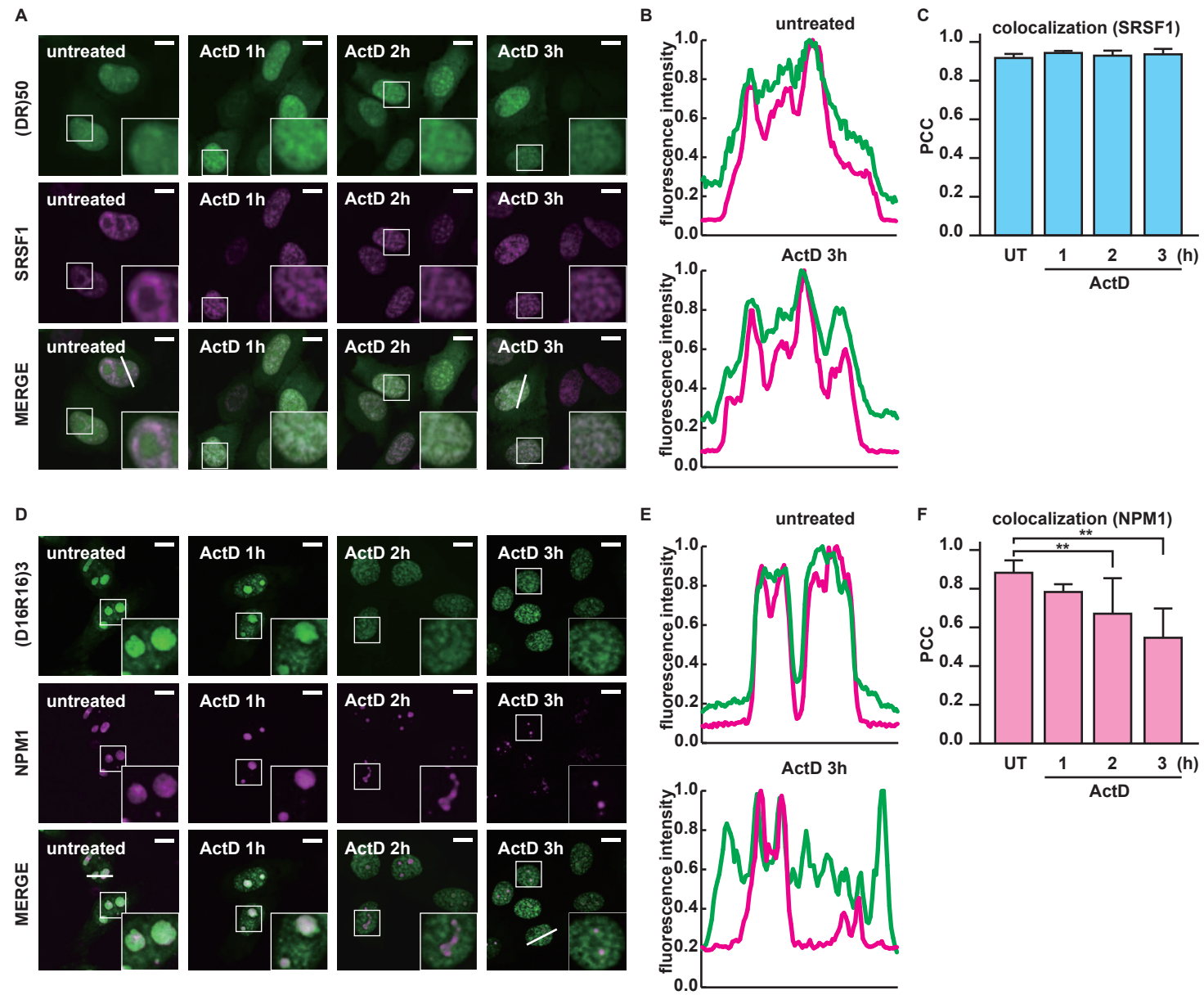

Supplement: Supplementary file 1 [file ijms-23-07658-s001.zip › Miyagi DR IJMS Fig.S2 070522.pdf]

Supplementary Figure S3

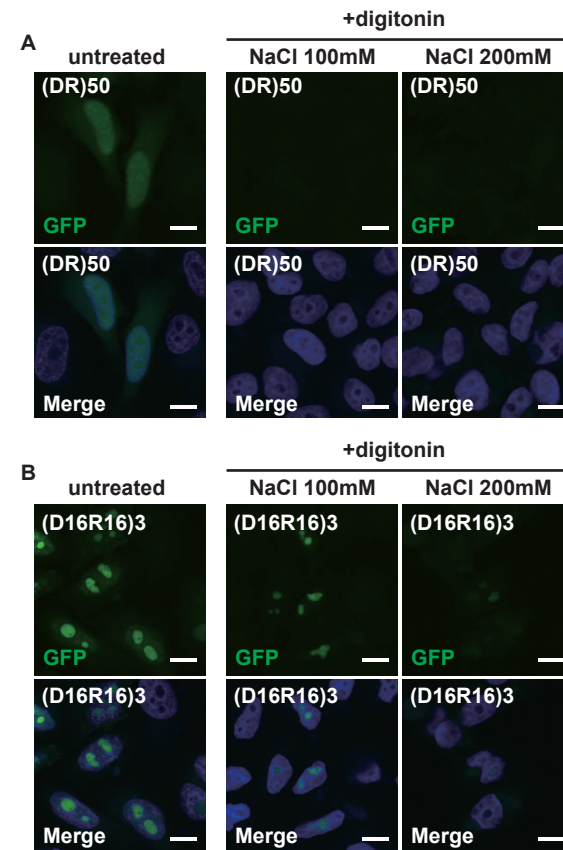

Supplement: Supplementary file 1 [file ijms-23-07658-s001.zip › Miyagi DR IJMS Fig.S3 070422.pdf]
